# Generative adversarial networks for construction of virtual populations of mechanistic models: simulations to study Omecamtiv Mecarbil action

**DOI:** 10.1007/s10928-021-09787-4

**Published:** 2021-10-29

**Authors:** Jaimit Parikh, Timothy Rumbell, Xenia Butova, Tatiana Myachina, Jorge Corral Acero, Svyatoslav Khamzin, Olga Solovyova, James Kozloski, Anastasia Khokhlova, Viatcheslav Gurev

**Affiliations:** 1 IBM Research, Yorktown, NY USA; 2 Ural Federal University, Yekaterinburg, Russia; 3 Institute of Immunology and Physiology, Ural Branch of the Russian Academy of Sciences (UB RAS), Yekaterinburg, Russia; 4 Department of Engineering Science, Institute of Biomedical Engineering, University of Oxford, Oxford, UK

**Keywords:** Omecamtiv Mecarbil, Populations of models, Generative adversarial networks, Parameter inference, Biophysical models

## Abstract

Biophysical models are increasingly used to gain mechanistic insights by fitting and reproducing experimental and clinical data. The inherent variability in the recorded datasets, however, presents a key challenge. In this study, we present a novel approach, which integrates mechanistic modeling and machine learning to analyze in vitro cardiac mechanics data and solve the inverse problem of model parameter inference. We designed a novel generative adversarial network (GAN) and employed it to construct virtual populations of cardiac ventricular myocyte models in order to study the action of Omecamtiv Mecarbil (OM), a positive cardiac inotrope. Populations of models were calibrated from mechanically unloaded myocyte shortening recordings obtained in experiments on rat myocytes in the presence and absence of OM. The GAN was able to infer model parameters while incorporating prior information about which model parameters OM targets. The generated populations of models reproduced variations in myocyte contraction recorded during in vitro experiments and provided improved understanding of OM’s mechanism of action. Inverse mapping of the experimental data using our approach suggests a novel action of OM, whereby it modifies interactions between myosin and tropomyosin proteins. To validate our approach, the inferred model parameters were used to replicate other in vitro experimental protocols, such as skinned preparations demonstrating an increase in calcium sensitivity and a decrease in the Hill coefficient of the force–calcium (F–Ca) curve under OM action. Our approach thereby facilitated the identification of the mechanistic underpinnings of experimental observations and the exploration of different hypotheses regarding variability in this complex biological system.

**Supplementary Information:**

The online version of this articlecontains supplementary material available 10.1007/s10928-021-09787-4.

## Introduction

Complex biological systems, such as tissues, exhibit tremendous cell-to-cell variability of biochemical and physical properties, which in turn underlie both stability and variability in the functioning of these systems. As a result, researchers face major difficulties in the design and analysis of experiments that attempt to provide conclusive evidence about the biological mechanisms underlying these variable observations. Computational models are increasingly leveraged to provide plausible, quantitative explanations of the observed phenomena, and this approach has become indispensable for hypothesis generation. However, these biophysical models are often also highly complex in their own right, making inference of model parameters that produce model outputs matching variable experimental data a major challenge and bottleneck in research design. Researchers in different areas have expended significant effort developing mechanistic models of complex biological systems to capture the variability in experimental measurements by constructing virtual populations of models, i.e. parametric families of models fit to groups of in vitro datasets.

“Population of models” has received significant attention in the cardiac modeling community over the last decade following initial developments in the field of neuroscience [31, 43]. This avenue of research has greatly improved understanding of model input-output relationships among cardiac models [51] and influenced the construction and refinement of biophysical models used to identify molecular mechanisms of arrhythmia and transition to disease, as well as to explore variability in response to drugs across different patient cohorts [5, 14, 21, 23, 34, 40, 41, 47, 50, 60]. In a recent study, Lawson et al. [23] proposed a general heuristic method for the construction of populations of models that employs Markov chain Monte Carlo (MCMC) methods to sample model parameters and reproduce biomarkers from the action potential of isolated human cardiomyocytes. Significant work has also been carried out in quantitative systems pharmacology to improve methods for generation and selection of virtual patient populations, which capture the statistics of clinical populations [3, 10, 12, 18, 46].

State-of-the-art methods to construct populations of models are essentially limited to finding a single distribution of model parameters *x* with density $q_X(x)$ that, after being pushed by a function $y = M(x)$ into the space of model outputs *y*, produces a distribution of features with density $q_Y(y)$ that matches experimental/clinical observations,

1 $$\begin{aligned} x \sim q_X(x)\; \Rightarrow y = M(x)\; \Rightarrow y \sim q_Y(y). \end{aligned}$$

For instance, among cardiac models of the action potential, features *y* could represent upstroke velocity, action potential duration, and other characteristics of the action potential shape. Parameters of the model, *x*, would typically represent conductivity of the membrane channels and transition rates of channel gates. Note that although most biophysical models are represented by systems of ordinary differential equations, the relationship between *x* and *y* can always be represented by the function $y=M(x)$, despite not always being used in the closed form. As the function $y=M(x)$ is typically not invertible, the problem in (1) is under-determined, and an infinite number of possible densities $q_X(x)$ of model parameters can provide a correct solution. The prior on the parameters $p_X(x)$ provides an additional constraint, which in its simplest form comprises a uniform distribution with upper and lower bounds for each parameter (possibly derived from observations in other experiments).

In real-world applications, the parameter inference problem may have a more complicated structure that is not easy to address with traditional approaches based on rejection sampling, prevalence weighting and MCMC methods [10, 23, 46]. For example, in the current study, we have in vitro cardiac mechanics data from two groups of myocytes, the control group and the group with presence of a cardiac inotrope Omecamtiv Mecarbil (OM). Our goal is to find parameters of the mechanistic model that reproduce the experimental observations from both groups. Currently, such parameter inference problems are usually solved independently for each group [23]. However, this independent treatment of each group may ignore additional constraints that come from prior knowledge about the biophysics involved. In our case, the prior knowledge comes from previous experiments showing that OM does not affect calcium concentration and cell stiffness, as it directly targets contractile proteins [13, 27]. Therefore, marginal distributions of parameters that are not affected by the drug must be the same for cell groups under the two different conditions, yet this requirement is impossible to satisfy if the groups are treated independently. It is tempting to attempt sequential analysis to infer parameters from a first group then fix their distribution for analysis of a second group. However, this approach fails due to its asymmetry, in that the distributions of the model parameters are dependent on the observations in the output space of the model.

Formally, the graphical forward model of the composite problem described above is expressed as

2 $$\begin{aligned} \begin{array}{llll} &{} x_{2,c} \sim q_{X_{2,c}|{X_1}}(x_{2,c} | x_1) \Rightarrow &{} y_c = M(x_c) = M(x_1, x_{2,c}) \Rightarrow &{} y_c \sim q_{Y_c}(y_c)\\ x_1 \sim q_{X_1}(x_1)\Rightarrow &{} &{} \\ &{} x_{2,d} \sim q_{X_{2,d}|{X_1}}(x_{2,d} | x_1) \Rightarrow &{} y_d = M(x_d) = M(x_1, x_{2,d}) \Rightarrow &{} y_d \sim q_{Y_d}(y_d) \end{array} \end{aligned}$$

Model parameter vectors $x_c=[x_1, x_{2,c}]$ for the control group and $x_d=[x_1, x_{2,d}]$ for the drug group comprise three parameter sets: $x_1$, which is not affected by the inotrope, $x_{2,c}$, made up of unmodified drug targets in the control group, and $x_{2,d}$, the same targets after the drug is applied. The model produces outputs $y_c$ and $y_d$ with distribution densities $q_{Y_c}(y_c)$ and $q_{Y_d}(y_d)$ matching the densities derived from the experimental data. Note that pairwise information such as joint distribution $q_{Y_c, Y_d}(y_c, y_d)$ is not available unless the experiment is conducted on the same group of cells in different conditions. Our goal is to infer joint densities of model parameters, $q_{X_c}(x_c) = q_{X_c}(x_1, x_{2,c})$ and $q_{X_d}(x_d) = q_{X_d}(x_1, x_{2,d})$ from given experimental observations $q_{Y_c}(y_c)$ and $q_{Y_d}(y_d)$.

A wide variety of parameter inference problems for populations of models exist, for which different requirements from prior biophysical knowledge must be satisfied, but current MCMC-based methods are limited to tackling simpler problems, as formulated in (1). Therefore, other statistical and machine learning methods are needed to solve these complex parameter inference scenarios. The deep learning field has experienced an explosion of novel method development in the last decade. Neural networks, such as normalizing flows for explicit density estimation, have been successfully applied in parameter inference problems for simulation-based inference [11]. Another generative neural network from the deep learning domain that can compete with MCMC is the Generative Adversarial Network (GAN) [15]. Several GANs have been proposed for variational inference and could replace MCMC methods for sampling from posterior distributions in general [17]. We have recently proposed a novel GAN [39] specifically for parameter inference problems as given in (1), which is readily extensible to incorporate estimation of $q_{X_c}(x_c)$ and $q_{X_d}(x_d)$ from experimental data $q_{Y_c}(y_c)$ and $q_{Y_d}(y_d)$ as in (2). This GAN architecture implements a constrained optimization formulation of the stochastic inverse problem and is designed to solve parameter inference problems with these difficult requirements using the complex structures it makes possible.

Here, we constructed populations of cardiac ventricular myocyte models using our novel GAN architecture to better understand the action of the OM drug, which has shown promising results in improving cardiac function in clinical trials [54, 56, 57]. Although in the late stages of clinical trials, the molecular mechanism underlying improved contractility by OM is still not well understood, and recently a phase III clinical trial with OM also missed some key endpoints [55]. A diverse set of experimental studies have revealed different actions of OM on cross-bridge (XB) cycling dynamics [2, 16, 19, 20, 25, 27, 28, 30, 36, 52, 59], indicating a need for improved understanding of OM mechanism of action. In particular, our population of myocyte models captured experimentally observed unloaded shortening contraction profiles recorded in isolated cell preparations in the control and OM groups. The calibrated population of myocyte models allowed us to characterize the variability inherent in the experimental data and map the observed changes in experimental signals after drug application to quantitative changes in only those mechanistic model parameters that our prior knowledge points to as being targeted by the drug. Our modeling results thereby provide a quantitative interpretation of experimental data. To validate our findings, we used the inferred model parameters to simulate a different experimental protocol in skinned cell preparations, which is commonly used to obtain the steady-state force-calcium (F-Ca) relationship. We are the first, to our knowledge, to qualitatively reproduce in simulation the most distinctive feature of OM: increased calcium sensitivity and a decrease in the Hill coefficient of the F-Ca curve.

## Methods

### Experimental methodology

#### Myocyte isolation

This study was carried out in accordance with the Directive 2010/63/EU and recommendations of The Animal Care and Use Committee of the Institute of Immunology and Physiology UB RAS. The experimental protocol was approved by The Animal Care and Use Committee of IIP. Studies were performed using healthy male Wistar rats (7 animals), 16-24 weeks of age. The rats were injected intramuscularly with heparin sodium (5000 U/kg) to prevent development of coronary thrombosis, anesthetized 20 min later with 30 mg/kg mg/kg tiletamine/zolazepam (Zoletil100®, Virbac, France) and 20 mg/kg xylazine hydrochloride (Alfasan, The Netherlands), and then in 15-20 min were subjected to a terminal bilateral thoracotomy for removal of the heart. Left ventricular cardiomyocytes were isolated using the combined Langendorff perfusion method and intraventricular injection technique with a collagenase-containing solution as described in detail elsewhere [7]. After perfusion of the isolated heart by enzymes to digest extracellular matrix using the Langendorff system, the heart was transferred in a Petri dish and perfused by intraventricular injections. Then, cells were removed from digestion buffer and resuspended in a sequence of perfusion buffers supplemented with fetal bovine serum and with gradually increasing calcium concentrations (0.1-1.0 mM). The cell suspension was transferred to Hepes-buffered Tyrode solution (in mM: NaCl 140, KCl 4.7, MgSO4 1.2, CaCl2 1.8, HEPES 10, glucose 11.1, pH of 7.4 adjusted using NaOH). Experiments were performed after allowing cells to rest for at least 40 minutes.

#### Unloaded sarcomere shortening measurements

Sarcomere shortenings were measured by a laser confocal scanning microscope (LSM 710, Carl Zeiss, Germany). Only rod-shaped cardiomyocytes with well-defined sarcomere striations were measured. Cardiomyocytes that did not respond to pacing were excluded regardless of appearance. Prior to the measurement, a narrow region of scanning was selected on the cell image and the intensity profile was recorded. Data signal was then processed and the mean sarcomere length was derived from the striation pattern based on Discrete Fourier Transform using custom made software [35].

#### Omecamtiv Mecarbil treatment

All chemicals except for collagenase (Collagenase Type 2, Worthington Biochemical Corporation, USA) and CK-1827452 (OM, Selleck Chemicals LLC) were obtained from Sigma-Aldrich Co. (St. Louis, MO, USA). Stock solution with final OM concentrations 10 mM were prepared in DMSO as solvent and stored at 4 ${^{\circ }}$C. Appropriate volume of the concentrated stock solution was dissolved in Tyrode solution to obtain final OM concentration of 1 $\mu$ M carried in 0.01 % DMSO. The control (OM-free) solution contained the same amount of DMSO (0.01 %). The quiescent cells from OM group were exposed to OM for 8 min and then were field-stimulated at 1 Hz at least 2 minutes for equilibration prior to functional measurements. The duration of OM treatment for each cell did not exceed more than 13 minutes All single cell experiments were performed at 36±1 ${^{\circ }}$C.

### Biophysical and statistical modeling

To simulate groups of cells in the presence and absence of OM, 1) a model of sarcomere contraction was fit to the data from the literature to obtain a default set of parameters for rat myocytes, 2) parameter sensitivity analysis was performed, selecting sets of parameters to simulate cell-to-cell variability, and 3) a generative model was trained to estimate density $q_{X_c}(x_c)$ and $q_{X_d}(x_d)$. More details on each of the 3 stages of analysis are given in the following sections.

**Fig. 1:**
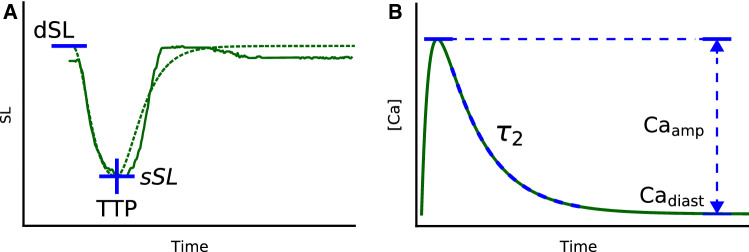
Feature extraction and parameters of calcium transient. **A** An example of fitting (3) (dotted green line) to the sarcomere length trace (solid green line) and extraction of *dSL*, *sSL*, and *TTP*. **B** Calcium transient in the model simulated with Eq. (6)

For our problem, $q_{Y_c}(y_c)$ and $q_{Y_d}(y_d)$ are densities of 3 features that were extracted from experimental traces of sarcomere shortening in the presence and absence of OM, respectively. The extracted features include diastolic length (*dSL*), systolic length (length at the peak of shortening) (*sSL*), and time to peak of sarcomere shortening (*TTP*) and were obtained by fitting the equation,

3 $$\begin{aligned} \begin{aligned} SL(t) = (dSL - sSL)\times (1 - e^{-log^2\left[ \frac{t}{TTP}\right] }) + sSL \end{aligned}, \end{aligned}$$

to the experimental traces (Fig. 1A). Here, *t* is the time variable; *SL*(*t*) is the change in the sarcomere length with time. Explicit density models $q_{Y_c}(y_c)$, $q_{Y_d}(y_d)$ were built for each group of cells by fitting a multivariate Gaussian.

### Model of sarcomere

We used our previously proposed phenomenological mean-field model of myofilament contraction [1] to evaluate the effects of different mechanisms of OM action. The myofilament model is a modified version of [45] with the addition of XB-XB cooperative effects and a simple mean-field strain formulation similar to [44]. The model equations were designed to reproduce several experimental features observed in both isolated muscle and physiological measurements at the ventricle level. The complete set of model equations is listed in the Supplemental Material. Here, we only discuss equations with parameters that will be inferred by the generative model.

$\mathbf{n }_\mathbf{A }$, $\mathbf{A }_\mathbf{50 }$. We simulated XB dynamics from the perspective of XB-groups, similar to previous models describing cooperative behavior of XBs within regulatory units (RUs) (e.g., [37]). The core model equation describes the formation and collapse of XB “populations”, groups of XBs featuring XB-XB cooperative effects within a group of a fixed size, with a fraction of groups $G_{XB}$ in the sarcomere:

4 $$\begin{aligned} \frac{d\mathrm {G}_{XB}}{dt} = f_G({\bar{A}})(1-\mathrm {G}_{XB}) - g_G({\bar{A}},s) \ \mathrm {G}_{XB}. \end{aligned}$$

Active tension $T_a = S_a \times \mathrm {G}_{XB} \times ...$ generated by the sarcomere is a product of several factors including a scaling constant $S_a$ and $G_{XB}$. Forward $f_G$ and reverse $g_G$ rates depend on average XB strain *s* and ${\bar{A}}$, the ratio of RUs in permissive states, when active sites are open for myosin heads, to RUs in nonpermissive states. The ratio ${\bar{A}}$ is controlled by the amount of troponin C (TnC) with bound calcium, tropomyosin chain properties among other factors. We describe the complex nonlinear relationship between ${\bar{A}}$ and the fraction *A* of troponin bound with calcium as a Hill equation:

5 $$\begin{aligned} {\bar{A}} = \frac{A^{n_A}}{A^{n_A} + A^{n_A}_{50}}. \end{aligned}$$

Our main assumption was that the primary effect of OM could be simulated by changing only parameters $n_A$ and $A_{50}$, thereby also satisfying the requirement that OM not affect calcium concentration and cell stiffness.

$\mathbf{Ca }_\mathbf{amp }$, $\mathbf{Ca }_\mathbf{diast }$, ${\varvec{\tau }}_\mathbf{2 }$. To simulate cell-to-cell variability, several other parameters of the model were selected (Fig. 1). These are amplitude of calcium transient $Ca_{amp}$, diastolic calcium $Ca_{diast}$ concentration, and the constant of exponential decay $\tau _2$ that define the shape of calcium transient. The calcium transient *Ca*(*t*) equation is defined as in [45],

6 $$\begin{aligned} Ca(t) = \frac{Ca_{amp} - Ca_{diast}}{\beta } (e^{\frac{-(t - t_{start})}{\tau _1}} - e^{\frac{-(t - t_{start})}{\tau _2}}) + Ca_{diast} \end{aligned}$$

where

7 $$\begin{aligned} \beta = (\frac{\tau _1}{\tau _2})^{\frac{-1}{\frac{\tau _1}{\tau _2} - 1}} + (\frac{\tau _1}{\tau _2})^{\frac{-1}{1 - \frac{\tau _1}{\tau _2}}}. \end{aligned}$$

For information on other parameters in equations (4)-(6) please refer to the Supplemental Material and our previous publication [1].

$\mathbf{passive }_{{\varvec{\alpha }}}$. In mechanically unloaded shortening, myocytes contract against their own stiffness. We define passive tension $T_p$ as

8 $$\begin{aligned} T_p = \left\{ \begin{array}{ll} passive_{\alpha } * (1 - e^{passive_{\beta } \times (1 - \lambda )}) &{} \lambda < 1\\ passive_{\alpha } * (e^{passive_{\beta } \times (\lambda - 1)} - 1) &{} \lambda \ge 1 \end{array}\right. , \end{aligned}$$

where $\lambda$ is the sarcomere stretch ratio. Exponential constant $passive_{\beta }= 10$ was fixed for simulations. Scaling factor $passive_{\alpha }$ was variable. Since for unloaded shortening we solved equation $T_p = T_a$, tension units could be canceled out, and we set $passive_{\alpha } = 1$ at $S_a = 2.5e+5 \mu m^{-1}$.

$\mathbf{SL }_\mathbf{slack }$. We made an assumption that residual forces generated by XBs are minimal at diastole of unloaded shortening in the control group and resting length could be considered as the slack length $SL_{slack}$ of the sarcomeres. Due to variability of $SL_{slack}$ in the experimental data, it was included as one of the variable parameters of the model. The original model has a fixed constant of the slack sarcomere length $1.9 \mu m$. Instead of modifying the model, we scaled output model length *SL* by the ratio of $SL_{slack}$ and $1.9 \mu m$. Thus, parameter $SL_{slack}$ did not need to be inferred by the generative model, and the distribution of $SL_{slack}$ was estimated directly from the data.

#### Global sensitivity analysis

We performed global sensitivity analysis to systematically examine the influence of model input parameters on various model-derived metrics. Mean Decrease Accuracy (MDA) was used for estimation of sensitivity, as in our previous study [38]. Briefly, sensitivity analysis via MDA requires fitting a linear or non-linear regression model (e.g., linear regression model, random forest regression model, etc.) between the input parameters and the model-derived metric. Once trained, the model is fixed, and the performance of the regression model (e.g., given by $R^2$ score) is re-evaluated on modified input datasets obtained by randomly shuffling value entries of each of the parameters one at a time. More details and results of the analysis are given in the Supporting Material.

### Parameter inference

**Fig. 2:**
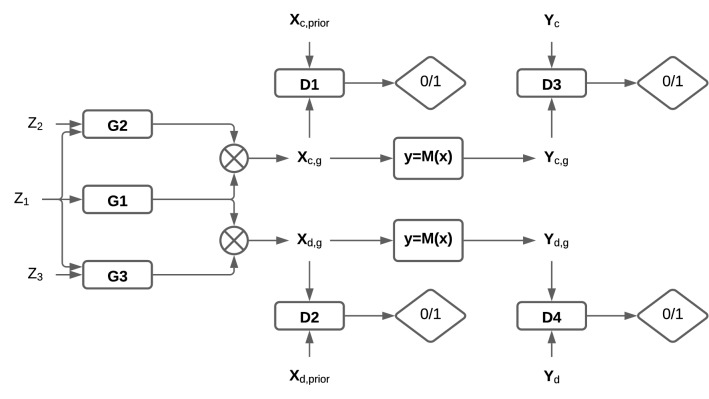
Generative network for model parameter inference. The generator network is trained to transform random variables $Z_1$, $Z_2$, and $Z_3$ with base Gaussian distributions to random variables with densities $q_{X_c,g}(x_{c,g})$ and $q_{X_d,g}(x_{d,g})$ as approximations of $q_{X_c}(x_c)$ and $q_{X_d}(x_d)$. The generator factorizes density by using 3 networks $G_1$, $G_2$, and $G_3$. The network $G_1$ is responsible for parameters $x_1$ that do not change under the drug action. $G_2$ is responsible for parameters that are affected by the drug $x_{2,c}$ and generates their values for the control group. $G_3$ is the same as $G_2$, but for the group under action of the drug. The conditional dependence of $x_{2,d}$ and $x_{2,c}$ on $x_1$ in (2) is implemented by the input of samples from the base distribution $Z_1$ for $G_1$ to both $G_2$ and $G_3$. Parameters are pushed through the model $y=M(x)$ to obtain $q_{Y_c,g}(y_{c,g})$ and $q_{Y_d,g}(y_{d,g})$ as approximations of $q_{Y_c}(y_c)$ and $q_{Y_d}(y_d)$. Discriminators $D_1$, $D_2$ separates samples $x_{c,g}$ and $x_{d,g}$ from samples of the prior distribution of the parameters (uniform in our case). $D_3$ and $D_4$ are discriminators for model outputs

A generative model was trained to fit a parametric family of sarcomere models to observations from two groups of myocytes. The generative model has a structure similar to generative adversarial networks (GAN) [15]. Standard GANs consists of two competing networks: the generator and discriminator. The goal of training the generator network is to transform samples from white noise to samples from a target distribution. The discriminator, structured as a classifier, is trained to distinguish between samples from a true target distribution and samples produced by the generator. In the Nash equilibrium between the generator and the discriminator, the generator samples represent an implicit density model of a target distribution. We extended standard GANs to a GAN with multiple discriminators and incorporated the mechanistic model into the network (Fig. 2). In the new setup, the generator is trained to produce samples from the distribution of mechanistic model parameters such that the distribution of model outputs is coherent with the target observations. Full details of the GAN network shown in Fig. 2 are provided in the Supporting Material.

To incorporate the model into the GAN, the function $y = M(x)$ should be given in a closed form and should be differentiable. However, the model of sarcomere is in the form of a system of ordinary differential equations (ODE). Although, it is possible to incorporate ODE systems into neural networks [9], integration of ODEs during GAN training would be computationally expensive. Instead, we incorporate a surrogate model of $y=M(x)$ into the GAN, constructed by training a feed-forward neural network on samples from the prior.

## Results

### Data recordings

**Fig. 3:**
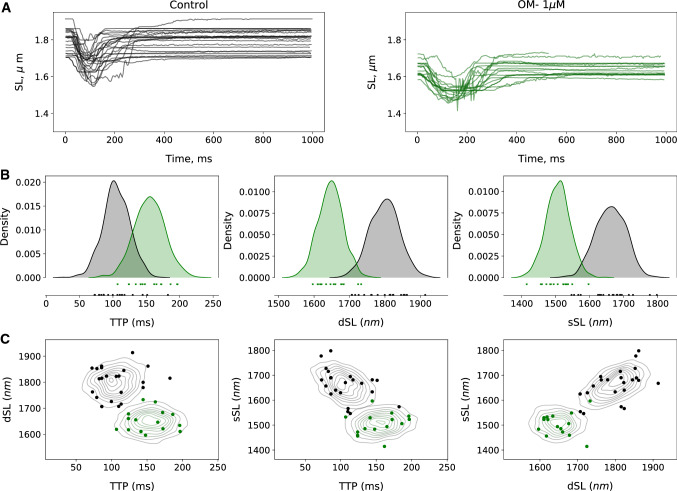
Raw length traces and feature density estimation. **A** Traces of unloaded shortening recorded from isolated myocytes in the control group (black solid lines) and in the group with OM (green solid lines). **B** Plot of estimated marginal distributions of the time-to-peak (*TTP*), resting/diastolic sarcomere length (*dSL*) and the sarcomere length at the peak of shortening (*sSL*) features approximated via multivariate Gaussian fit to the data. **C**. Estimated joint density distribution of the features in the presence (black contours) and absence of OM (green contours). The solid circles in **C** indicate the experimental data points

The unloaded sarcomere shortening traces in the control group (*n* = 24 cells) and OM group (*n* = 17 cells) are plotted in Fig 3A. Contraction of myocytes in the OM group (1 $\mu$M) are characterized by a longer duration of contraction and a shift in the diastolic (resting length) of the sarcomeres. The sarcomere length traces were fitted with equation (3). The *sSL*, *dSL* and *TTP* features were extracted for all myocytes. The distributions of the extracted features for the control and OM groups were approximated by multivariate Gaussians. Fig 3B shows the estimated marginal distribution of these features. The presence of OM resulted in an increase in mean *TTP* by approximately 50 ms. The mean of the *SL* and *dSL* features decreased by about 0.2 $\mu m$. The pairwise joint densities of the features are shown in Fig 3C.

### Parameter inference

**Fig. 4:**
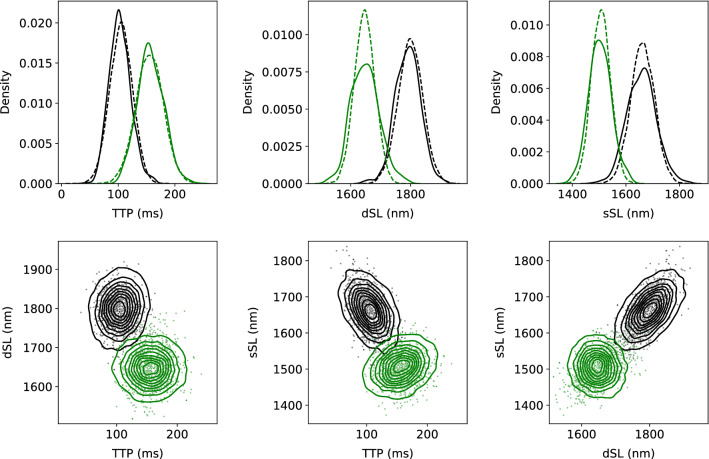
Features generated by populations of models. **Top row**. Plot of marginal distributions produced by the generative model (solid lines) against real data (dashed lines) for the time to peak (*TTP*), diastolic sarcomere length (*dSL*) and the sarcomere length at the peak of shortening (*sSL*) in control (black lines) and OM (green lines) groups. **Bottom row**. Joint density distributions of the observed features in control (black contours) and OM (green contours) groups. Points are produced by the generative model

The generative model was trained with a uniform prior on model parameters with ranges $Ca_{amp}$: [0.5, 2], $Ca_{diast}$: [0.05, 0.4], $\tau _2$: [20, 140], $n_A$: [2, 20], $A_{50}$: [0.4, 1.5], and $passive_{\alpha }$: [0.05, 7]. After training, 10,000 $x_c$ and $x_d$ samples were run through the model to obtain $y_c$ and $y_d$. As shown Fig 4, the generative model was able to reproduce the distribution of features present in the experimental data.

**Fig. 5:**
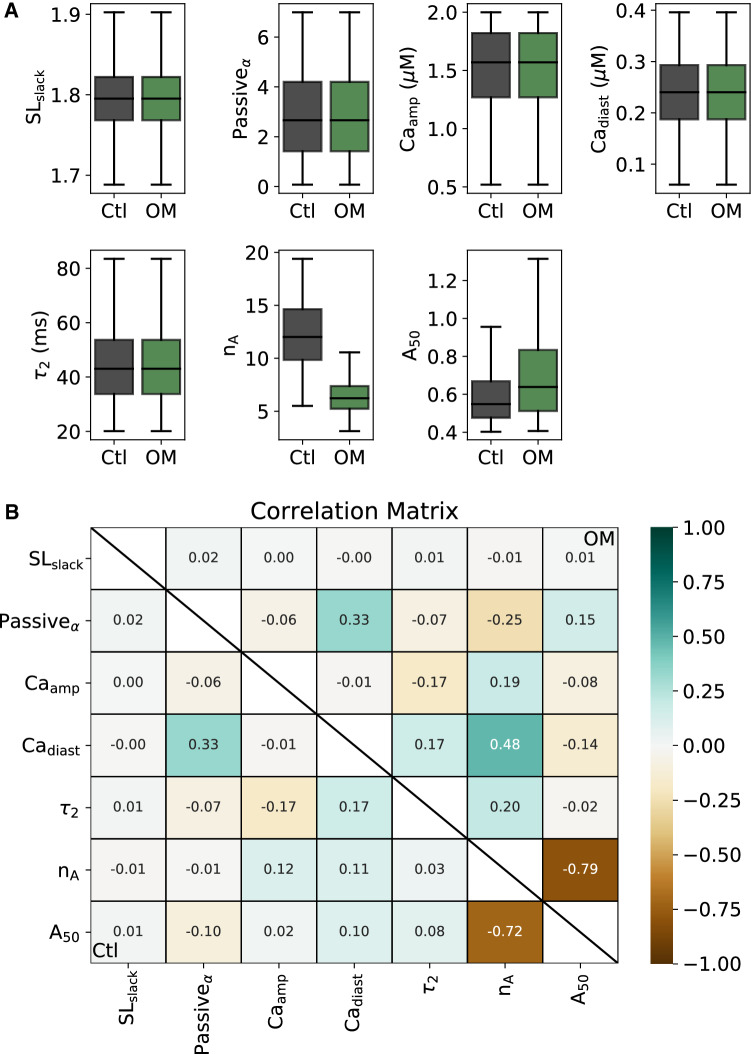
Distribution of model parameters from the generative model. **A** Plot of marginal distributions of model parameters produced by the generative model in the control (black) and OM (green) groups. **B** Correlation matrix of model parameters. Lower triangle of the matrix is correlation coefficients of parameters for myocytes in the control group, upper triangle for OM group

Densities of inferred parameters $x_c$ and $x_d$ are shown in Fig 5. The marginal distribution for each parameter and the correlation matrices for both $x_c$ and $x_d$ (Fig 5B lower-left and upper-right triangles, respectively) are shown to characterize the inferred parameter distributions. OM decreases the mean of the Hill coefficient $n_A$ and slightly increases $A_{50}$ in the dependency between a fraction of thin filament sites accessible for myosin heads and a fraction of TnC with bound calcium. In the sarcomere model, decreasing $n_A$ indirectly reduces the Hill coefficient of the F-Ca relationship, as we demonstrate below, while $A_{50}$ affects calcium sensitivity.

The strongest correlation between parameters of the model is observed between $n_A$ and $A_{50}$ (Fig 5B). In general, there are two reasons why these inferred parameters might correlate. First, a strong correlation could result between two redundant parameters when both parameters have a similar effect on the model output. Second, the correlation could result from coupling between parameters of a specific physical process. In our case, $n_A$ and $A_{50}$ have different effects on the dynamics of sarcomere contraction, and therefore their coupling explains the correlation. Encouragingly, the correlations between most other parameters are weak, as the generative model captures the independence of different mechanisms underlying the myocyte contraction.

### Simulations of isosarcometric (F–Ca) and isometric curves

Next, we examined if the effect of OM, represented in the generative model by variation and correlation in the parameters $n_A$ and $A_{50}$, was sufficient to capture not only the unloaded shortening data but also other in vitro tests of OM’s effects previously reported in the literature [19, 36, 49]. We took the means of the inferred model parameters for control and OM groups and used these to simulate the isosarcometric and isometric curves.

**Fig. 6:**
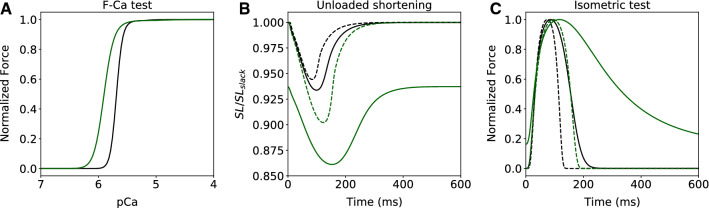
**Effects of OM on isosarcometric and isometric curves and link with diastolic length of unloaded shortening.** **A** F–Ca relationship for model with mean parameters for control (black) and OM group (green). **B** Unloaded contraction for the same mean parameters as in **A** for $Ca_{diast} = 0.2 \mu M$ (solid lines) and $Ca_{diast}=0$ (dashed lines). **C** Isometric contraction for the same mean parameters as in **A** for $Ca_{diast} = 0.2 \mu M$ (solid lines) and $Ca_{diast}=0$ (dashed lines)

In the isosarcometric contraction protocol, intracellular calcium concentration is kept constant at different levels in skinned preparations held at constant length and steady state force *F* generated by a cell is registered to build the F-Ca relationship. The F-Ca curve is usually fit with a Hill equation to quantify calcium sensitivity,

9 $$\begin{aligned} F = \frac{Ca^h}{Ca^h + Ca^h_{50}}, \end{aligned}$$

and here we use notation *Ca* for calcium concentration, *h* for the Hill coefficient, and calcium sensitivity as $Ca_{50}$ that denote calcium concentration at the half of the maximal *F*. In Fig 6A, the concentration of calcium is expressed as the negative log ($pCa = -log(Ca)$). F-Ca curves for two parameter sets taken as an average of parameters for the control and OM groups are shown in Fig 6A. The Hill coefficient *h* decreases from 8.15 in the control to 4.99 in the OM group. $Ca_{50}$ decreases from $2.06 \mu M$ to $1.25 \mu M$. Thus, the model qualitatively captures one of the key features of OM action. Indeed, the decrease of F-Ca slope and increase in calcium sensitivity was consistently reported in multiple publications [19, 36]. Changes in F-Ca also explain the shorter diastolic length of the sarcomeres in the OM group during unloaded shortening experiments. The simulations indicate that the level of diastolic intracellular calcium is sufficient to activate filaments and produce the residual force. In Fig. 6B (solid lines), we show results from simulating unloaded shortening with average parameters for each cell group, then setting $Ca_{diast}=0$ and repeating the simulations (Fig 6B dashed lines). A decrease in end-diastolic length due to OM action does not appear in the simulations with zero diastolic calcium levels. The unloaded shortening simulations also nicely captured a distinctive feature of OM action, which is that the shortening velocities of sarcomeres do not change in presence of the drug. This feature also appears in simulations of contraction of the whole ventricle that are presented in the Supplemental Material.

Figure 6C shows simulated force traces for the two parameter sets taken as an average of parameters for the control and OM groups under isometric conditions (i.e. fixed sarcomere lengths at 10% stretch and transient increases in Ca). The simulation results show that OM significantly increases the time from peak contraction to 50% relaxation as observed experimentally in force responses measured in human engineering myocardium under increasing concentrations of OM [49]. The diastolic calcium levels can alter the OM induced increase in time from peak contraction to 50% relaxation as show in Fig. 6C.

## Discussion

Multi-modal data is typically gathered across different phases of drug-development. Mechanistic models serve as tools for analysis and interpretation of experimental data, to guide therapeutic design and gain improved understanding of mechanisms of action of the drug. A critical step towards application of mechanistic models for supporting drug design and clinical studies is identification of model parameters that provide model outputs consistent with the data. Biological data always presents itself with inherent variability. It is surprisingly common to handle this variability by simply averaging the characteristics/features derived from the data then finding a single set of model parameters in the model-based analysis. Even without variability, non-invertibility of the models creates uncertainty, and finding a single set of parameters is insufficient for proper analysis. In this study, we have addressed these limitations by developing new methods and applying them to analysis of in vitro experiments with a cardiac inotrope. Two groups of isolated myocytes were compared in experiments with unloaded shortening, the control group and the group of cells under the action of Omecamtiv Mecarbil. The main novelty of the study is an application of a novel generative model for parameter inference based on Generative Adversarial Networks. We also provided a model-based interpretation of experimental findings.

### Populations of models

The populations of models approach allows deterministic biophysical models to capture and explain the inherent variability present in biological data. The approach allows for identification of not only the best model (i.e., parameter set) but a population of parameter sets with a distribution that results in model outputs consistent with the data. Population-based modeling has gained increasing traction over the last decade in the fields of cardiac, neuroscience and quantitative systems pharmacology modeling [10, 21, 31, 34, 41, 43, 46, 47, 51]. To handle non-linearity among complex multiscale models, a recent study has proposed a general heuristic framework that uses a combination of sequential Monte Carlo (SMC) methods and a simulated annealing-type algorithm for constructing populations of models [23]. These current methods, however, have been limited to finding a distribution of model parameters for a single group. Even in scenarios in which data is available for treated and untreated groups, virtual population construction and parameter inference are carried out independently for each group [23]. Inferring parameters by treating groups independently imposes minimal constraints and ignores available prior knowledge about the underlying biophysics. For example, in the current work, we knew that OM directly targets contractile proteins and does not alter myocyte calcium dynamics or its stiffness.

In the early stages of analysis, we attempted to use existing methods from populations of models based on the theoretical inferential framework proposed by Poole and Raftery [42]. However, due to limitations of these existing methods, we found it difficult to utilize some of the prior biophysical knowledge such as that the treatment modulates only a subset of model parameters. Therefore, we developed the new GANs-based generative model, which can handle complex simulation scenarios and incorporate prior biophysical knowledge in different forms. Similar to graphical models, this generative model incorporates different prior information by factorization of distribution densities that are produced after modifying a structure of the GAN generator. The new generative model also allows fitting of multiple marginal distributions (e.g, $q_{Y_c}(y_c)$ and $q_{Y_d}(y_d)$) in cases when pairwise data (e.g, joint distribution $q_{Y_c, Y_d}(y_c, y_d)$) for cells in control and drug conditions are not available. Moreover, we have shown previously [39] that even for standard scenarios, the proposed GAN architectures can replace standard Bayesian inference methods [6, 42] and thereby allow construction of populations of deterministic and non-deterministic models coherent to the data for a single population. Standard conditional GANs could also be used for parameter inference, providing the advantage of amortized inference and its computationally fast parameter sampling [39]. Challenges in the adoption of GANs include unreliable training outcomes due to problems such as mode collapse, which several approaches have aimed to resolve [58]. A limitation in our use of GANs derives from the requirement that the mechanistic model must be differentiable for calculation and backpropagation of gradients in a Deep Learning architecture. However, mechanistic simulators often solve differential equations numerically, and therefore cannot be incorporated directly. Our solution was to use a differentiable surrogate model in place of the mechanistic simulation (see Methods). However, this approach can introduce bias if the target region of interest is under-sampled during training. To alleviate bias, active learning in simulation based inference studies [26] performs sequential refinement of conditional density model training data to iteratively improve the surrogate model.

#### Model-based interpretation of OM action

OM is known to induce a positive inotropic effect by selectively targeting cardiac myosin proteins without inducing alterations in the cytosolic calcium transient [16, 27]. Previous studies have also revealed that OM has little effect on the myocyte electrophysiological properties except at supratherapeutic concentrations [13, 16, 53]. Hence, we considered the scenario wherein the action of OM is captured by only a subset of model parameters. The parameters of the calcium transients ($Ca_{amp}, Ca_{diast}, \tau _2$) and slack length ($SL_{slack}$) and stiffness of cell ($passive_{\alpha }$) were assumed to be subject to cell-to-cell variability, while at the same time having the same distributions between the control and drug cells groups.

In sarcomeres, attachment of myosin heads to a thin filament is triggered by binding of calcium to troponin. Due to complex physical properties of tropomyosin chains, which open active sites on actin monomers for myosin heads in response to calcium binding, the relationship between the fraction of accessible active sites and concentration of calcium-TnC is nonlinear and affected by multiple factors, simulated in many modeling studies (e.g., [22, 33]). In our model, we represent this relationship as the Hill equation (5) and hypothesized that the effect of OM could be expressed by altering the parameters $n_A$ and $A_{50}$ of the Hill equation. Indeed, we successfully reproduced several key features of OM action that are apparent in experiments. First, OM has minimal effect on unloaded shortening velocity (Fig. 6B). This a distinct feature of OM action and is especially evident in comparisons of OM with other compounds that increase the amplitude of the calcium transient, such as isoproterenol [27]. Second, OM increases sensitivity to calcium at low calcium concentrations and decreases the Hill coefficient of the F–Ca relationship (Fig. 6A), as reported consistently across experiments [19, 28, 36, 52]. This effect is translated to the whole ventricles, as OM increases ejection time without significant changes in $dP/dt_{max}$ (see the Supplemental Material). Moreover, we also observed that OM significantly increases the time from peak contraction to 50% relaxation in isometric tests (Fig. 6C) as observed experimentally [49].

One possible interpretation of OM induced increase in calcium sensitivity and reduced Hill coefficient in our simulations is that OM prolongs the strong-bound state of XBs in agreement with previous experimental findings [25, 27, 28]. Strong-bound XBs can interact with tropomyosin to keep it in the open state in the absence of bound calcium resulting in thin filament activation, represented by the effect we captured by modifying parameters $n_A$ and $A_{50}$ of (5) in our phenomenological mean-field model. Further testing of this interpretation requires constructing and testing stochastic models of thin filament activation. Recently, a simple two-state stochastic model of thin filament activation was employed by Woody et al. [59] to support the experimental hypothesis of OM induced prolonged attachment of myosin heads to activate thin filaments. Their model [59] reproduced the observed changes in sensitivity and maximum force reported in [36], but does not capture the decrease in the Hill-coefficient of the F-Ca curve. Our group has put significant effort into developing, analyzing and testing new and existing stochastic models to reproduce the evading effect (results not shown). Our initial analysis suggests that altering XB-XB cooperativity in such stochastic models either has no effect on the Hill-coefficient or has the opposite effect (i.e., an increase in Hill-coefficient with increased calcium sensitivity) to the one observed experimentally. Further analysis with stochastic models is needed to clarify this matter.

Alternatively it is possible that the effect of OM is not due to cooperative XB-XB interaction through tropomyosin but due to other modulatory effects of OM on XBs that directly increase the probability of XB binding at previously unfavorable tropomyosin configurations. Indirect evidence that supports this explanation comes from experiments with mutations in cardiac tropomyosin proteins. Unlike OM, tropomyosin mutations alter the probabilities of myosin binding to actin by affecting the tropomyosin properties rather than affecting myosin. The functional effect of the tropomyosin mutations on the F–Ca relationship, however, is similar to OM induced effects. Increases in calcium sensitivity with simultaneous reduction in Hill coefficient have been reported in experiments with tropomyosin mutations [4, 24, 48]. Our interpretation of the model findings leads to the hypothesis that OM directly modifies the interaction between myosin heads and tropomyosin, thus increasing the probability that myosin heads attach to active sites that were previously inaccessible in closed states of tropomyosin. This hypothesis could be tested by examining OM induced effects in myocardial preparations with mutant tropomyosin.

There are other possible key contributors regulating OM action that we did not consider in the model. Cardiac myosin-binding protein C (cMyBPC), which is known to directly interact with myosin, can play a key role in regulating the inotropic effect of OM, as shown recently [29]. At submaximal calcium levels, OM-mediated force enhancements were significantly diminished in myocardial preparations with nonphosphorylatable cMyBPC. OM induced recruitment of myosin from the SRX state, as suggested in [19], can result in increased calcium sensitivity and enhanced force generation at low calcium levels. Moreover, cMyBPC can play a role in regulating OM induced recruitment of myosin from the SRX state, as it has also been shown to specifically disrupt myosin in the SRX state [32]. Stochastic models with explicit SRX state or mean-field models as in [8], which consider myosin in different “ON” and “OFF” states (SRX), need to be analyzed in order to better understand the possible effects of OM on the myosin SRX state.

In closing, our method using GANs for parameter estimation could be further applied to enhanced models, such as those proposed with stochastic mechanisms, whose aim is to further elucidate tests of our hypothesis and estimate uncertainties due to ambiguity in model parameters. These models would then capture the observed effects and reproduce experimental observations, as our mean field model has when coupled with GANs. We also note that these results for OM could not have been achieved using standard methods such as MCMC, which would have only allowed sampling parameters independently from the two groups and resulted in different distributions of parameters, including different distributions of the fixed parameters depending on the order of sequential sampling. We therefore propose that the use of our approach is currently the only path to solving a broad family of problems including the one solved here. Finally, we note that the statistical model in this paper is based on a Deep Learning solution, i.e., GANs, and that a more general formulation [39] could be extended to other statistical models for explicit or implicit density estimation. Such advanced tools for parameter inference have the potential to enable rapid adoption of in silico trials across a wide variety of applications because of their powerful treatment of prior information in different populations.

## Supplementary Information

Below is the link to the electronic supplementary material.

Electronic supplementary material 1 (PDF 1419 kb)
