# Supplementary material for: Generative adversarial networks for construction of virtual populations of mechanistic models: simulations to study Omecamtiv Mecarbil action
Source: J Pharmacokinet Pharmacodyn. 2021 Oct 29;49(1):51–64. doi: 10.1007/s10928-021-09787-4 (PMC8837558; doi:10.1007/s10928-021-09787-4)
Supplement: Supplementary file 1 — Electronic supplementary material 1 (PDF 1419 kb) [file 10928_2021_9787_MOESM1_ESM.pdf]

Noname manuscript No.
(will be inserted by the editor)

Supplemental Material - Generative Adversarial Networks for construction of virtual populations of mechanistic models: Simulations to study Omecamtiv Mecarbil action

Jaimit Parikh[1], · Timothy Rumbell [1], · Xenia Butova [3], · Tatiana Myachina [3], · Jorge Corral Acero [4], · Svyatoslav Khamzin [3], · Olga Solovyova [2,3], · James Kozloski [1], · Anastasia Khokhlova [2,3], · Viatcheslav Gurev [1*] ·

Myofilament contraction model

We have recently proposed a phenomenological mean-field model of myofilament contraction [1], which is employed in the current study for simulating and understanding the action of the inotrope Omecamtiv Mecarbil. We briefly describe here the model structure, but for further details, please refer to our earlier work [1]. The model is a modified version of [9], with the addition of XB-XB cooperative effects, and with a simple mean-field strain formulation similar to [8]. The model equations were designed to reproduce several experimental features observed in both isolated muscle and physiological measurements at the ventricle level.

We simulated XB dynamics from the perspective of XB-groups. The core model equation describes the formation and collapse of XB "populations", groups of XBs featuring XB-XB cooperative effects within a group. The equation for the fraction of groups G_{XB} engaged in force generation is

$$\frac{d\mathrm{G}_{XB}}{dt} = f_G(A)(1 - \mathrm{G}_{XB}) - g_G(A, s)\ \mathrm{G}_{XB}, \tag{1}$$

where f_G and g_G rates are both functions of the fractions of troponin complexes with bound calcium (A), and g_G is also a function of the mean XB strain (s). The fraction of bound XBs (XB_G) within a group is described as

$$\frac{d\mathrm{XB}_G}{dt} = f_{XB}(1 - \mathrm{XB}_G) - g_{XB}(s)\ \mathrm{XB}_G, \tag{2}$$

where the rate f_{XB} is a constant parameter, and g_{XB} is a function of the XB strain. The total fraction of XBs in the force-generating state is equal to $G_{XB} \times XB_G$. The rates f_G and g_G were formulated as functions of A,

$$\begin{aligned} f_G\left(A\right) &= \bar{f}_G \left(\frac{A^{n_A}}{A^{n_A} + A_{50}^{n_A}}\right)^{\zeta}, \\ g_G\left(A, s\right) &= min\left\{ g_G(s) \left(\frac{A^{n_A}}{A^{n_A} + A_{50}^{n_A}}\right)^{\zeta - 1}, g_{Gmax} \right\}, \end{aligned} \tag{3}$$

where $0 \leq \zeta \leq 1$ and $\bar{f}_G$ are model parameters, g_G is a function of XB strain, and A_{50} and n_A are parameters that define cooperativity in the interaction between troponin, tropomyosin state, and crossbridge dynamics in the XB-groups. The equation for the mean XB strain is,

$$\frac{ds}{dt} = \frac{1}{2}\frac{dSL}{dt} - f_{XB}\frac{1 - \mathrm{XB}_G}{\mathrm{XB}_G}s, \tag{4}$$

where SL is the sarcomere length. The rates g_{XB} and g_G in (2) and (3) are functions of s.

$$\begin{aligned} g_{XB}\left(s\right) &= \bar{g}_{XB} \times s_{mod}\left(s\right) \\ g_G\left(s\right) &= \bar{g}_G \times s_{mod}^{\eta}\left(s\right), \end{aligned} \tag{5}$$

* Corresponding Author: Viatchelslav Gurev, E-mail: vgurev@us.ibm.com
[1] IBM Research, Yorktown, NY, USA
[2] Ural Federal University, Yekaterinburg, Russia
[3] Institute of Immunology and Physiology, Ural Branch of the Russian Academy of Sciences (UB RAS), Yekaterinburg, Russia
[4] Institute of Biomedical Engineering, Department of Engineering Science, University of Oxford, Oxford, UK

where x_0 is the distortion due to XB power-stroke, and $\bar{g}_G$, $\bar{g}_{XB}$, α, and η are parameters of the model.

The length dependence for the thick filament is described by a piecewise polynomial function

$$\mathrm{LDF}_{\mathrm{thick}}\left(\lambda\right) = 0 \vee \left[\mathrm{LDF}_{\mathrm{thickmax}} \wedge \left(\lambda_{\mathrm{ms0}} \times \left(\lambda - \lambda_{\mathrm{mn0}}\right)\right) \wedge \left(\lambda_{\mathrm{ms1}} \times \left(\lambda - \lambda_{\mathrm{mn1}}\right) + \mathrm{LDF}_{\mathrm{thickmax}}\right)\right], \tag{6}$$

where $\wedge$ and $\vee$ are **min** and **max** binary operators, respectively, λ is the stretch ratio of a sarcomere (we assume a sarcomere length of 1.9 μm at $\lambda = 1$), $\lambda_{\mathrm{ms0}} > \lambda_{\mathrm{ms1}}$ are the slopes of the piecewise linear function, and $\lambda_{\mathrm{mn0}} < \lambda_{\mathrm{mn1}}$ are the nodes defining the function discontinuities. The active tension developed by the muscle (T_a) is then calculated as

$$T_a = S_a \times \mathrm{LDF}_{\mathrm{thick}}\left(\lambda\right) \times G_{XB} \times XB_G \times (s + x_0), \tag{7}$$

where T_a is the active tension; S_a is the scaling factor for the tension; x_0 is the step size of the myosin power stroke. Cardiac troponin complexes modulate myofilament contraction in a Ca-dependent manner by regulating the availability of myosin binding sites on actin. This is captured as in [9],

$$\frac{d}{dt}\mathrm{A}_H = k_{\mathrm{onT}}[\mathrm{Ca}](1 - \mathrm{A_H}) - k_{\mathrm{offHT}}\mathrm{A}_H, \tag{8}$$

$$\frac{d}{dt}\mathrm{A}_L = k_{\mathrm{onT}}[\mathrm{Ca}](1 - \mathrm{A}_L) - k_{\mathrm{offLT}}\mathrm{A}_L, \tag{9}$$

$$\mathrm{A} = \mathrm{A}_H \times \mathrm{LDF}_{\mathrm{thin}}\left(\lambda\right) + \mathrm{A}_L \times \left(1 - \mathrm{LDF}_{\mathrm{thin}}\left(\lambda\right)\right), \tag{10}$$

where A_H and A_L refer to the fraction of high and low calcium affinity troponin sites, respectively, that have Ca bound to their regulatory binding sites; k_{onT} is the rate constant for binding; [Ca] is the concentration of Ca; k_{offHT} and k_{offLT} are the rate constants for unbinding from the high and low affinity sites, respectively. Similar to the thick filament, a function for length dependence is defined for the thin filament as

$$\mathrm{LDF}_{\mathrm{thin}}\left(\lambda\right) = 0 \vee \left[\mathrm{LDF}_{\mathrm{thinmax}} \wedge \left(\lambda_{\mathrm{as0}} \times \left(\lambda - \lambda_{\mathrm{an0}}\right)\right) \wedge \left(\lambda_{\mathrm{as1}} \times \left(\lambda - \lambda_{\mathrm{an1}}\right) + \mathrm{LDF}_{\mathrm{thickmax}}\right)\right]. \tag{11}$$

The equation for intracellular calcium transient that drives the contraction is

$$\beta = \left(\frac{\tau_1}{\tau_2}\right)^{-1/\left(\frac{\tau_1}{\tau_2}-1\right)} - \left(\frac{\tau_1}{\tau_2}\right)^{-1/\left(\frac{\tau_2}{\tau_1}-1\right)}, \tag{12}$$

$$[Ca]\left(t\right) = \begin{cases} Ca_{diast},\ t \le t_{start} \\ \left(\frac{Ca_{amp} - Ca_{diast}}{\beta}\right) \times \left(e^{-\frac{t - t_{start}}{\tau_1}} - e^{-\frac{t - t_{start}}{\tau_2}}\right) + Ca_{diast},\ t > t_{start} \end{cases}, \tag{13}$$

where τ_1 is the time constant for the upstroke of the calcium transient, τ_2 is the time constant for the decay of the calcium transient, Ca_{diast} represent the diastolic calcium level, and Ca_{amp} is the amplitude of the calcium transient. The summary of the model equations is listed below,

Myofilament model equations

$$\beta = \left(\frac{\tau_1}{\tau_2}\right)^{-1/\left(\frac{\tau_1}{\tau_2}-1\right)} - \left(\frac{\tau_1}{\tau_2}\right)^{-1/\left(\frac{\tau_2}{\tau_1}-1\right)}$$

$$[Ca]\,(t) = \begin{cases} Ca_d,\ t \le t_{start} \\ \left(\frac{Ca_{amp}-Ca_{diast}}{\beta}\right) \times \left(e^{-\frac{t-t_{start}}{\tau_1}} - e^{-\frac{t-t_{start}}{\tau_2}}\right) + Ca_{diast},\ t > t_{start} \end{cases}$$

$$SL = 1.9\mu m \times \lambda$$

$$s_{mod} = e^{-\alpha(s/x_0)^2}$$

$$f_G\,(A) = \bar{f}_G \left(\frac{A^{n_A}}{A^{n_A} + A_{50}^{n_A}}\right)^{\zeta}$$

$$g_G\,(s) = \bar{g}_G \times s_{mod}^{\eta}$$

$$g_{XB}\,(s) = \bar{g}_{XB} \times s_{mod}$$

$$g_G\,(A, s) = min\left\{ g_G(s)\left(\frac{A^{n_A}}{A^{n_A} + A_{50}^{n_A}}\right)^{\zeta-1}, g_{Gmax}\right\}$$

$$\frac{d\mathrm{G}_{XB}}{dt} = f_G(A)(1 - \mathrm{G}_{XB}) - g_G(A,s)\ \mathrm{G}_{XB}$$

$$\frac{d\mathrm{XB}_G}{dt} = f_{XB}(1 - \mathrm{XB}_G) - g_{XB}(s)\ \mathrm{XB}_G$$

$$\frac{ds}{dt} = \frac{1}{2}\frac{dSL}{dt} - f_{XB}\frac{1 - \mathrm{XB}_G}{\mathrm{XB}_G}s$$

$$\frac{d}{dt}\mathrm{A}_H = k_{\mathrm{onT}}[\mathrm{Ca}](1 - \mathrm{A_H}) - k_{\mathrm{offHT}}\mathrm{A}_H$$

$$\frac{d}{dt}\mathrm{A}_L = k_{\mathrm{onT}}[\mathrm{Ca}](1 - \mathrm{A}_L) - k_{\mathrm{offLT}}\mathrm{A}_L$$

$$\mathrm{A} = \mathrm{A}_H \times \mathrm{LDF_{thin}}\,(\lambda) + \mathrm{A}_L \times (1 - \mathrm{LDF_{thin}}\,(\lambda))$$

$$\mathrm{LDF_{thick}}\,(\lambda) = 0 \vee [\mathrm{LDF_{thickmax}} \wedge (\lambda_{\mathrm{ms0}} \times (\lambda - \lambda_{\mathrm{mn0}})) \wedge (\lambda_{\mathrm{ms1}} \times (\lambda - \lambda_{\mathrm{mn1}}) + \mathrm{LDF_{thickmax}})]$$

$$\mathrm{LDF_{thin}}\,(\lambda) = 0 \vee [\mathrm{LDF_{thinmax}} \wedge (\lambda_{\mathrm{as0}} \times (\lambda - \lambda_{\mathrm{an0}})) \wedge (\lambda_{\mathrm{as1}} \times (\lambda - \lambda_{\mathrm{an1}}) + \mathrm{LDF_{thinmax}})]$$

$$T_a = S_a \times \mathrm{LDF_{thick}}\,(\lambda) \times G_{XB} \times XB_G \times (s + x_0)$$

Default model parameter set

The myofilament model parameters were optimized to reproduce different *in vitro* experimental tests, such as the rate of force redevelopment (Ktr) test (Fig 1A), isosarcometric test (Fig 1B), unloaded shortening test (Fig 1C) and isometric test (Fig 1D), typically carried out to characterize myofilament properties as shown in Fig 1. The default model parameters are listed in Table 1 and were optimized to match *in vitro* experiments performed in rat myocyte preparations. The model simulations reproduced the steep increases of Ktr between low and high calcium concentrations and produced maximum Ktr values on the order of 60 s^{-1} (Fig 1A), as observed experimentally [2,13]. The model simulations also showed the typical cooperative relationship between force and calcium concentrations (i.e., significant increase in maximum force for small changes in calcium levels) (Fig 1B) upon stimulation at fixed sarcomere lengths and Ca concentration. The simulations also captured the experimentally observed [2] increases in maximal steady force and calcium sensitivity at higher sarcomere lengths. Finally, the model simulation produced increases in isometric force at larger sarcomere lengths (Fig 1D).

Parameter	Value	Bounds	Units
λ_{an0},λ_{mn0}	0.631579	-	unitless
λ_{an1},λ_{mn1}	1.21053	-	unitless
λ_{as0}	1.58333	-	unitless
λ_{as1}	0.791667	-	unitless
$\mathrm{LDF}_{\mathrm{thickmax}}$	1	-	unitless
λ_{ms0}	2.45161	-	unitless
λ_{ms1}	1.22581	-	unitless
$\mathrm{LDF}_{\mathrm{thinmax}}$	0.645833	-	unitless
k_{onT}	60	[10, 70]	$\mu M^{-1}s^{-1}$
k_{offHT}	60	[5, 70]	s^{-1}
k_{offLT}	900	[20, 1400]	s^{-1}
A_{50}	0.8	[0.4, 1.2]	unitless
n_A	10	[3, 15]	unitless
f_{XB}	26	[1, 50]	s^{-1}
$\bar{g}_{XB}$	100	[1, 50]	ms^{-1}
$\bar{f}_G$	260	[5, 1500]	ms^{-1}
$\bar{g}_G$	0.04	[5e-3, 5e-2]	ms^{-1}
Ca_{amp}	1	[0.5, 2]	μM
τ_1	23.07	-	ms
τ_2	190.20	[20, 140]	ms
Ca_{diast}	0.2	[0.05, 0.4]	μM
S_a	250×10^{-6}	-	$N\mu m^-3$
α	0.05 (positive strain)<br>1 (negative strain)		

Table 1 Myofilament model parameters.

Feature	Test
Time to peak (ttp_iso)	Isometric test
Time to 90% decay (ttd_iso)	Isometric test
Maximum/Peak force (max_iso)	Isometric test
Minimum rate of force redevelopment (ktr_min)	Ktr test
Maximum rate of force redevelopment (ktr_max)	Ktr test
Maximum force (fmax)	Isosarcometric (F-Ca) test
Hill coefficient (n) of the F-Ca curve	Isosarcometric (F-Ca) test
Calcium sensitivity (ca50) of the F-Ca curve	Isosarcometric (F-Ca) test
Time to maximum contraction (ttp)	Unloaded shortening test
Sarcomere length at rest (dSL)	Unloaded shortening test
Sarcomere length at maximum contraction (sSL)	Unloaded shortening test
Rate of sarcomere shortening (k1)	Unloaded shortening test
Rate of sarcomere relaxation (k2)	Unloaded shortening test

Table 2 Features extracted from simulations of isometric, isosarcometric, unloaded and rate of force redevelopment tests in the myocyte model

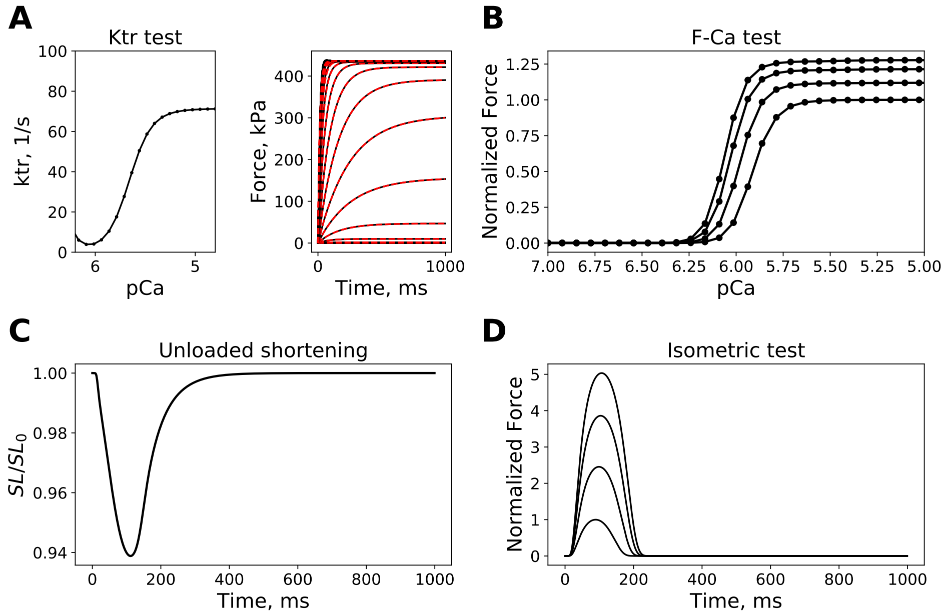

Fig. 1 Simulation results from the myofilament model. (**A**) Rate of force redevelopment at different calcium levels in the left panel. Force redevelopment after release-stretch at different Ca levels (black solid lines) fitted by mono-exponential curves (red dashed lines) are shown in the right panel. (**B**) Force traces during isosarcometric contraction for rat cardiomyocytes at sarcomere lengths (SL) equal to 1.9, 1.99, 2.05, and 2.09 μm normalized by the peak of force at slack length. (**C**) Example of unloaded shortening trace generated by the myofilament model. The sarcomere length (SL is normalized by the slack length (SL_0) (**D**) Force traces during isometric contraction for rat cardiomyocytes at sarcomere lengths equal to 1.9, 1.99, 2.05, and 2.09 μm normalized by the peak of force at slack length.

Regression model-based sensitivity analysis

Model performance is sensitive to random permutations of important parameters. We used Python's scikit-learn package [6] to train and test several different machine-learning models. The sensitivity results reported in Results were calculated with a random forest regression model. Estimation of sensitivity via MDA of the random forest regression model provides accurate measures even in presence of a nonlinear relationship between model inputs and outputs. Here, we provide comparison of the sensitivity estimates obtained using the linear regression model as in [11]. In order to determine the sensitivity estimates via MDA, we sampled 200, 000 random sets of model input parameters using Latin hypercube sampling (LHS). The parameter bounds considered for the LHS sampling are reported in Table 1. For each of the 200, 000 parameter sets, we simulated different sarcomere contraction tests typically carried out *in vitro* to characterize the myofilament properties. In particular, we simulated the unloaded shortening test, isometric test, isosarcometric test, and rate of force redevelopment test, then extracted features from these simulated tests (Table 2). Next, the random forest regression model was trained to predict each of the model output features as a combination of the model input parameters. Finally, the sensitivity measures were estimated via MDA.

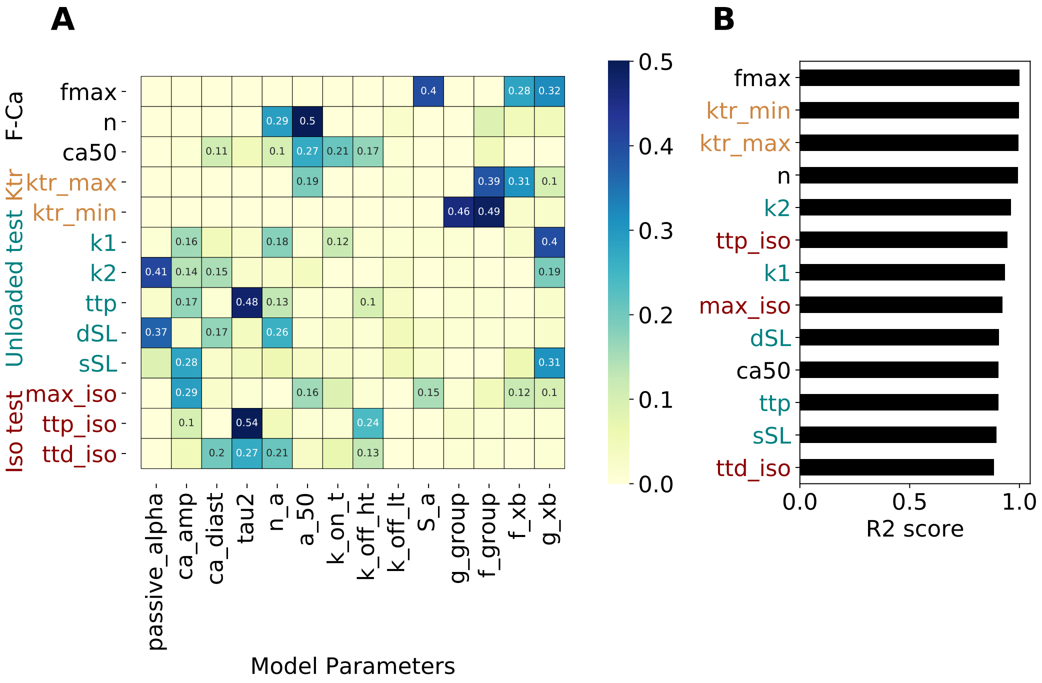

Fig. 2 Sensitivity of model features to perturbations in the model input parameters estimated via Mean Decrease in Accuracy (MDA) method. (**A**) Heatmap of sensitivity estimates , i.e., decrease in performance of the regression model upon random shuffling of the model input parameters obtained via the MDA method. (**B**) R2 score of the multivariate random forest regressor fit for the all features.

GAN configuration and training

The GAN architecture shown in Fig. 3 was used to train 3 generators to sample model parameters coherent with the experimental data in both control and OM conditions. To stabilize training and help prevent mode collapse, we also incorporated a reconstruction network that takes generated parameter samples concatenated from all 3 generators as input, and aims to reproduce the 3 sets of base variables $\boldsymbol{z}$. This approach is similar to that used in VEEGAN [12], but simplified, as it includes only the ℓ_2 component of the reconstructor loss, excludes the cross-entropy term and removes the dependence of the discriminator on $\boldsymbol{z}$.

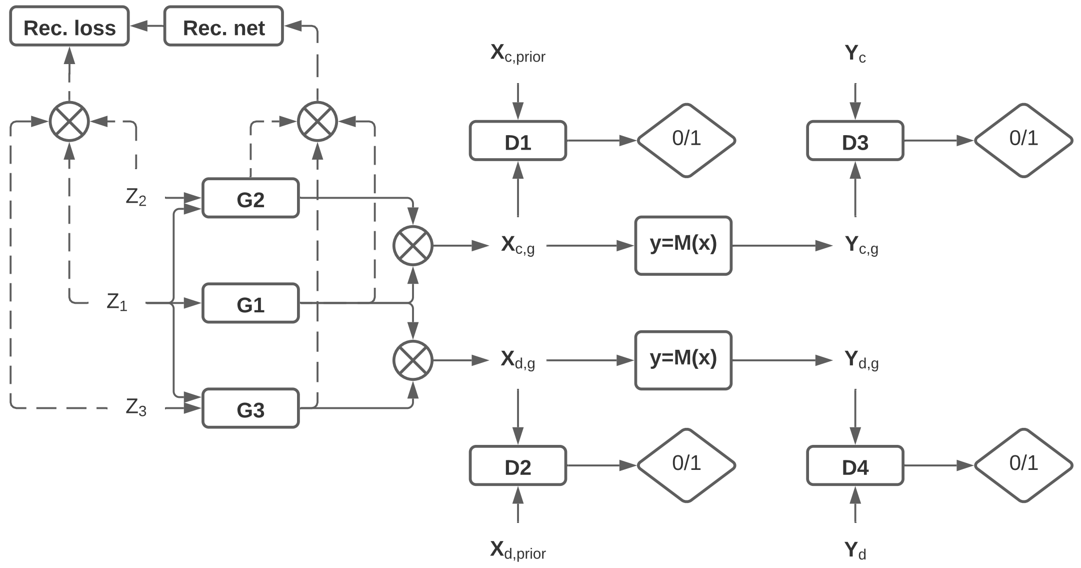

Fig. 3 Generative network for model parameter inference. The generator network is trained to transform random variables Z_1, Z_2, and Z_3 with base Gaussian distributions to random variables with densities $q_{X_c,g}(x_{c,g})$ and $q_{X_d,g}(x_{d,g})$ as approximations of $q_{X_c}(x_c)$ and $q_{X_d}(x_d)$. The generator factorizes density by using 3 networks G_1, G_2, and G_3. The network G_1 is responsible for parameters x_1 that do not change under the drug action. G_2 is responsible for parameters that are affected by the drug $x_{2,c}$ and generates their values for the control group. G_3 is the same as G_2, but for the group under action of the drug. The conditional dependence of $x_{2,d}$ and $x_{2,c}$ on x_1 is implemented by the input of samples from the base distribution Z_1 for G_1 to both G_2 and G_3. Parameters are pushed through the model $y = M(x)$ to obtain $q_{Y_c,g}(y_{c,g})$ and $q_{Y_d,g}(y_{d,g})$ as approximations of $q_{Y_c}(y_c)$ and $q_{Y_d}(y_d)$. Discriminators D_1, D_2 separates samples $x_{c,g}$ and $x_{d,g}$ from samples of the prior distribution of the parameters (uniform in our case). D_3 and D_4 are discriminators for model outputs. The additional structure of reconstruction network is added for GAN stabilization.

GAN loss functions In Fig. 3, discriminators $D3$ and $D4$ (termed D_Y) distinguish between samples from the distribution Y_c and Y_d and samples generated by the generators $G1$, $G2$ and $G3$ forwarded through the mechanistic model $y = M(x)$, for which the standard loss

$$L_{D_Y} = \mathbb{E}_{y\sim\mathcal{Q}_Y} \log[D_Y(y)] + \mathbb{E}_{z\sim\mathcal{P}_Z}, \log[1 - D_Y(M(G(z)))] \tag{14}$$

is maximized. Discriminators $D1$ and $D2$ (termed D_X) distinguish between samples from the prior over mechanistic parameters $X_{c,prior}$, $X_{d,prior}$ and samples generated by $G1$, $G2$ and $G3$, for which the standard loss

$$L_{D_X} = \mathbb{E}_{x\sim\mathcal{P}_X} \log[D_X(x)] + \mathbb{E}_{z\sim\mathcal{P}_Z} \log[1 - D_X(G(z))], \tag{15}$$

is maximized. The reconstruction network R aims to reproduce the original base distribution Z from samples generated by G, for which the squared loss is calculated

$$L_R = \mathbb{E}_{z\sim\mathcal{P}_Z} ||z - R(G(z))||^2. \tag{16}$$

The generator network G generates mechanistic parameter sets from the base variable Z, for which losses are calculated from all D_Y and D_X according to

$$\begin{aligned} L_{G_Y} &= \mathbb{E}_{z\sim\mathcal{P}_Z} - \log[1 - D_Y(M(G(z))] + \mathbb{E}_{z\sim\mathcal{P}_Z}, \log[D_Y(M(G(z)))], \\ L_{G_X} &= \mathbb{E}_{z\sim\mathcal{P}_Z} - \log[1 - D_X(G(z)] + \mathbb{E}_{z\sim\mathcal{P}_Z}, \log[D_X(G(z))]. \end{aligned} \tag{17}$$

The total loss for G is then the weighted sum loss

$$L_G = w_Y L_{G_Y} + w_X L_{G_X} + w_R L_R, \tag{18}$$

which is minimized, where $w_Y = 1.0$, $w_X = 0.1$, and $w_R = 1.0$ are used as default weights.

We used the Adam optimizer with step size of 0.0001 for G and R, and 0.00002 for D_X, and D_Y. The β_1 and β_2 parameters of the Adam optimizer were set to default values of 0.9 and 0.999, respectively, as suggested in [5]. Mini-batch size was 100. Training was performed in two stages. First, G, R and D_X were trained together, with $w_X = 1.0$ and the L_{D_Y} term removed in (18) (i.e. $w_Y = 0$), for 200 epochs to initialize G, where the parameter prior targets $X_{c,prior}$ and $X_{d,prior}$ were sampled $10,000$ times during each epoch. Second, the full GAN was trained for 400 epochs, also using $10,000$ target samples during each epoch for Y_c and Y_d, generated from the multivariate Gaussian fits to the experimental data. The neural networks used for each component in the architecture were all dense, feedforward networks, configured as shown in Table 3.

Table 3 Neural networks used in GAN architecture.

Network	Hidden layers	Nodes per layer	Dropout rate	Activation function
D_X	8	80	0.01	ReLU
D_Y	8	80	0.01	ReLU
G	8	160	0.0	ReLU
R	8	80	0.0	ReLU

Surrogate model

In this study, the mechanistic model described in the Myofilament model equations section, and represented by $\boldsymbol{y} = M(\boldsymbol{x})$, is not differentiable and is therefore challenging to incorporate into a deep learning network. Here, we trained a surrogate model in the form of a feedforward network to approximate $\boldsymbol{y} = M(\boldsymbol{x})$ for use as the model nodes in the GAN architecture. The surrogate network consisted of 4 dense layers with 400 nodes per layer, ReLU activation and a dropout rate of 0.1 between layers. We performed mechanistic model simulations using a Latin hypercube sampling of the 6 dimensional parameter space, calculating output features for $140,000$ parameter sets to provide a surrogate training dataset. To train the surrogate, we held out 20% of the dataset as test data, and trained the neural network for 100 epochs. Fig. 4 shows that the prediction performance of a trained surrogate on the test data was good, with the surrogate accurately approximating the mechanistic model outputs.

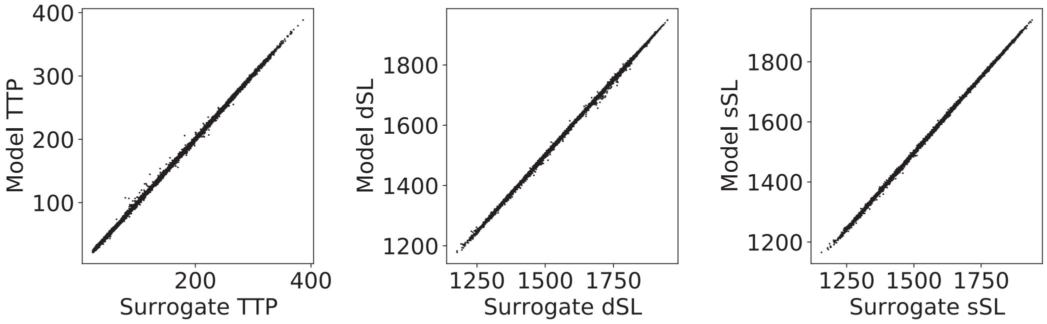

Fig. 4 Features Y predicted by a trained surrogate model from test data input parameter sets (x-axes, 'Surrogate') plotted against those simulated from the mechanistic model equations (y-axes, 'Model'). The surrogate models accurately approximated the function $\boldsymbol{y} = M(\boldsymbol{x})$.

GAN reproducibility testing

To test whether the results produced by the trained GAN architecture were robust given different trained surrogates or initial conditions, we performed 5 trials of GAN training, once using a different surrogate model across each trial, and once with a different random seed across each trial. The surrogates were trained using the same network structure, training data, and method, but will exhibit differences in network weights after training, and therefore some differences in predicted feature values, which will influence the GAN training. Fig. 5 demonstrates that the results we obtained were robust to deviations caused by the use of different surrogate models. Similarly, different initial conditions will influence the final trained weights of the GAN networks, even with the same surrogate model, so we tested different initial conditions using different random seeds. Fig. 6 shows that the parameters sampled by the trained GAN were also robust to differences in initial conditions.

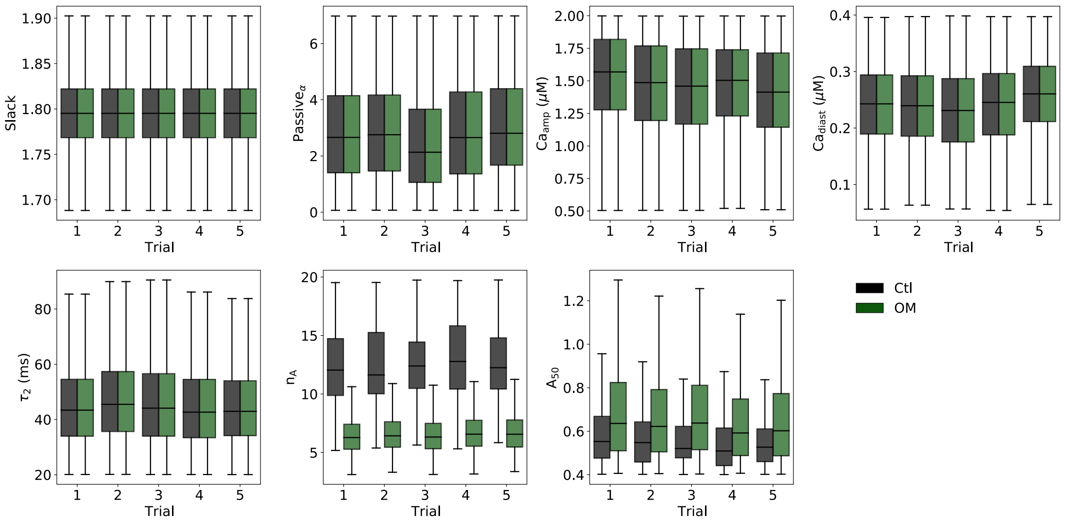

Fig. 5 Effect of multiple surrogates on parameters sampled by the GAN. Each 'trial' used a different surrogate model, trained using the same method and training data. Similar marginal distributions of parameters were sampled across trials for both control and OM conditions, indicating that the GAN training was robust to changes in the surrogate model.

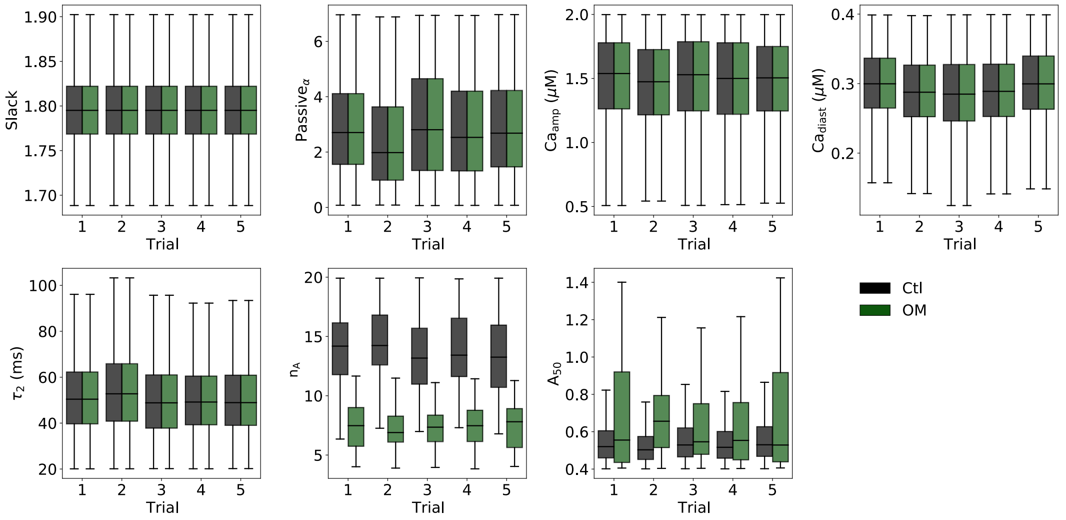

Fig. 6 Effect of multiple different random seeds on parameters sampled by the GAN. Each 'trial' used an identical surrogate model, but a different random seed for initialization of weights in the GAN. Similar marginal distributions of parameters were sampled across trials for both control and OM conditions, indicating that the GAN training was robust across trials with different initial conditions.

Effect of OM on left ventricular contraction

In previous work, we demonstrated an efficient method of training a low order model of ventricular mechanics via congruency training to reproduce the global behavior of a 3-D finite element model by scaling the outputs of just a single modeled cell [1]. Briefly, the low order models comprise the myofilament model coupled with a transformation module whose parameters are sized by machine learning to match the low order model outputs to features of the detailed finite element models. We demonstrated that simple linear transformations between sarcomere strain (tension) and ventricular volume (pressure) were sufficient to reproduce global pressure-volume outputs of 3-D finite element models. Here, we demonstrate our first results from simulations of OM with the low order model of ventricular mechanics. Although the results are preliminary, we include them here to outline a pipeline for translation of *in vitro* experiments into predictions of whole organ behavior.

Models of left ventricle

In our pipeline, we translated the *in vitro* simulation results described in this paper into the hemodynamics of the left ventricle (LV), simulating the effect of OM on the population of healthy (N) and heart failure (HF) hearts. For these purposes, geometric information based on echocardiographic data was extracted from retrospective medical records from healthy subjects (n=97) and patients affected by heart failure (n=261).

As in [3], left ventricular geometry was parameterized with 6 parameters: the outer radius at base Rb, the length of the longitudinal semi-axis of the outer spheroid Z, the ventricular wall thicknesses at base L and apex H, the sphericity/conicity of the spheroid $e \in [0, 1]$, and the truncation angle Ψ_0. In the first step, we used a regression model to obtain 6 parameters that describe LV geometry based on several echocardiographic features: end-diastolic volume (EDV), end-diastolic diameter, septal wall thickness, and lateral wall thickness. The publicly available SunnyBrook CMR dataset [7] was employed to train the linear regression:

1. The parameters Rb and Z were reformulated by subtracting L and H, respectively, in order to decouple these variables and minimize their dependency,
2. All geometry was standardized in volume (100mL) via similarity transformations to remove ventricle size variability. This standard volume was obtained by multiplying the parameters L, Rb, Z and H by the cubic root of the ratio between the target and the actual volume. The dimensionless parameters Ψ_0 and e were preserved.
3. A multivariate linear model was trained to estimate Z, H, Ψ_0 and e based on L and Rb, assuming that there is no intra-correlation among dependent variables (diagonal covariance matrix). The R2 correlation scores obtained were 0.79, 0.43, 0.34 and 0.09, respectively. However, e, the least correlated variable, can be determined instead by the volume constraint and after L and Rb are known and Z, H, Ψ_0 have been estimated.

The LV model was coupled with a Windkessel model of blood circulation, as in [1]. We fit the LV model parameters S_a and P_{at} to ensure an optimal match with the EDV and end-systolic volume (ESV). S_a and P_{at} represent the overall efficiency of contraction and a simplified representation of atrial pressure, respectively. To adapt parameters of the Windkessel model and achieve a central aortic blood pressure within the normal range of 100-120 mmHg, the hemodynamic parameters of the 3-element Windkessel model, i.e., aortic valve resistance R_1, aortic resistance R_2, distal resistance R_3 and capacitance C, were scaled proportionally to the stroke volume of each patient. The optimization problem was solved with the L-BFGS-B algorithm. All other parameters were taken from [1]. Average mismatches to the target variables were smaller than 2%.

To reproduce the OM effect, we changed the parameters n_A and A_{50} as shown in Table 4. Comparison analyses were carried out with respect to the pressure and volume traces, computed for the optimal set of parameters of each patient.

Table 4 The model parameters used for reproducing Omecamtiv Mecarbil's effect on whole organ behavior

	n_A	A_{50}
Baseline	10	0.32
Low concentration of OM	8.7	0.335
High concentration of OM	7.3	0.36

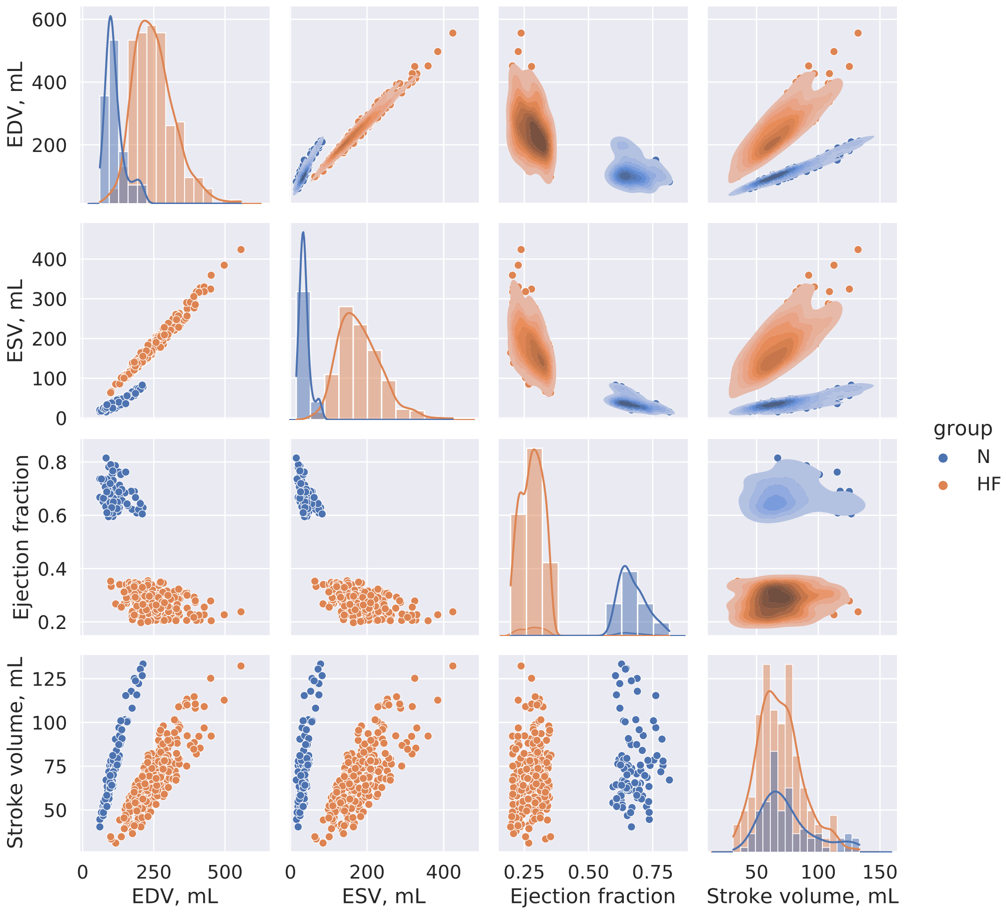

Fig. 7 Virtual population for a testing OM effects. The main indices of the ventricular population for N and HF cases are shown in blue and orange, respectively.

LV simulations

Fig. 7 shows simulated global outputs after the model parameters were optimized to match ESV and EDV for the N (blue color) and HF (orange color) cases. As expected, optimized values of the S_a parameters were significantly lower (mean -46%) for the HF compared to N. Optimized values of P_{at} parameters did not show differences (mean -5%) for HF compared to N. As shown in Fig. 7, the groups are divided by indices and correspond to the observed features in HF and N cases.

Fig. 8 shows traces demonstrating the contractile effect of simulated OM (dashed lines) for a low (left) and high (right) concentration of OM, compared to representative simulated HF traces (solid lines). As observed, OM decreased ESV and EDV but did not affect dP/dt. This results shows that OM's inotropic effect is limited by the impairment in diastolic function.

Fig. 9 shows changes from baseline to low (blue) and high (orange) concentrations of OM. The top row shows that the main effect of OM on left ventricular function is associated with a decrease in EDV and ESV. The difference between the HF and N groups is related to the size of the ventricles. Changes in ejection fraction between groups are the same, and changes in stroke volume are greater for HF compared to N (middle row). The bottom row shows that OM increases the ejection time for HF more than for N.

Our results are highly consistent with those obtained by other groups in animals and humans [4, 10]. For example, the results in pigs [10] show similar pressure-volume loops and the same changes in the main indices. Simulation results are quantitatively closer to clinical trials, and show the same changes of LV indices [4].

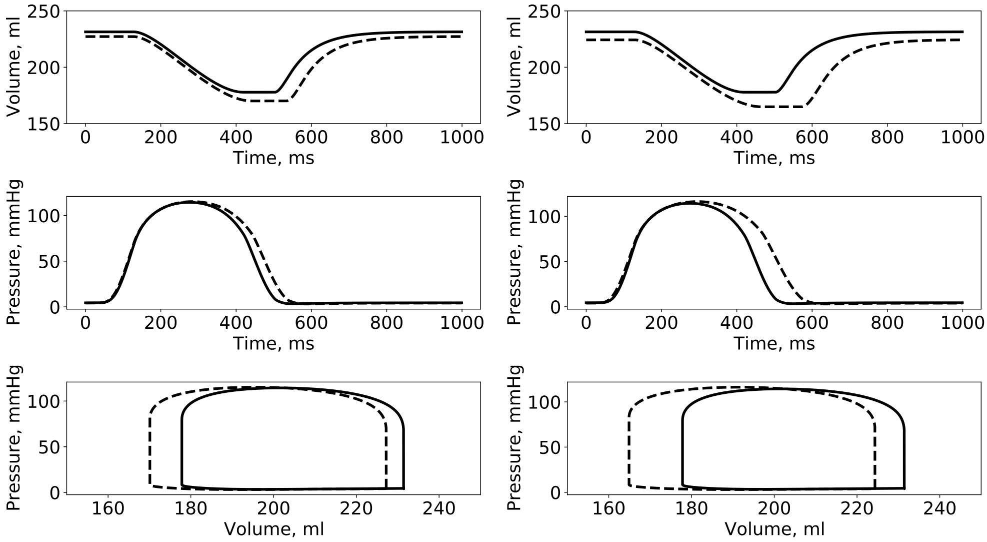

Fig. 8 Contractile effects of OM for representative HF case. Top row, simulated ventricular volume traces for the low (left) and high (right) concentration of OM. Middle row, corresponding simulated intraventricular pressure traces. Bottom row, simulated pressure-volume loop. This figure shows the correspondence of the results of modeling and experiments on animals.

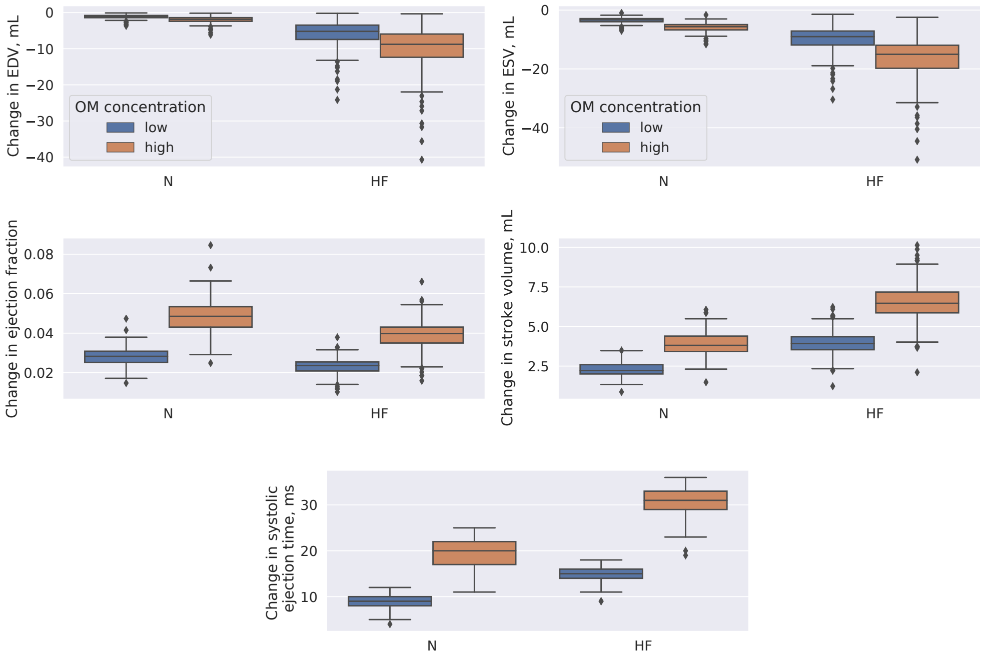

Fig. 9 Changes in heart indices induced by OM. Top row, changes in EDV and ESV for low (blue) and high (orange) concentration of OM.

References

1. Achille, P.D., Parikh, J., Khamzin, S., Solovyova, O., Kozloski, J., Gurev, V.: Model order reduction for left ventricular mechanics via congruency training. PLOS ONE **15**(1), e0219876 (2020). DOI 10.1371/journal.pone.0219876
2. de Tombe, P.P., Stienen, G.J.M.: Impact of temperature on cross-bridge cycling kinetics in rat myocardium. The Journal of Physiology **584**(Pt 2), 591–600 (2007). DOI 10.1113/jphysiol.2007.138693
3. Di Achille, P., Harouni, A., Khamzin, S., Solovyova, O., Rice, J.J., Gurev, V.: Gaussian Process Regressions for Inverse Problems and Parameter Searches in Models of Ventricular Mechanics. Frontiers in Physiology **9** (2018). DOI 10.3389/fphys.2018.01002
4. Edgardo, K., Mallarkey, G.: Cardiac myosin activators for heart failure therapy: focus on omecamtiv mecarbil. Drugs in context **7**, 212518 (2018)
5. Kingma, D.P., Ba, J.: Adam: A method for stochastic optimization. arXiv preprint arXiv:1412.6980 (2014)
6. Pedregosa, F., Varoquaux, G., Gramfort, A., Michel, V., Thirion, B., Grisel, O., Blondel, M., Müller, A., Nothman, J., Louppe, G., Prettenhofer, P., Weiss, R., Dubourg, V., Vanderplas, J., Passos, A., Cournapeau, D., Brucher, M., Perrot, M., Édouard Duchesnay: Scikit-learn: Machine learning in python (2012)
7. Radau, P., Lu, Y., Connelly, K., Paul, G., Dick, A., Wright, G.: Evaluation framework for algorithms segmenting short axis cardiac mri. The MIDAS Journal-Cardiac MR Left Ventricle Segmentation Challenge **49** (2009)
8. Razumova, M.V., Bukatina, A.E., Campbell, K.B.: Stiffness-distortion sarcomere model for muscle simulation. Journal of Applied Physiology (Bethesda, Md.: 1985) **87**(5), 1861–1876 (1999). DOI 10.1152/jappl.1999.87.5.1861
9. Rice, J.J., Wang, F., Bers, D.M., de Tombe, P.P.: Approximate model of cooperative activation and crossbridge cycling in cardiac muscle using ordinary differential equations. Biophysical Journal **95**(5), 2368–2390 (2008). DOI 10.1529/biophysj.107.119487
10. Rønning, L., Bakkehaug, J.P., Rødland, L., Kildal, A.B., Myrmel, T., How, O.J.: Opposite diastolic effects of omecamtiv mecarbil versus dobutamine and ivabradine co-treatment in pigs with acute ischemic heart failure. Physiological Reports **6**(19), e13879 (2018). DOI https://doi.org/10.14814/phy2.13879. URL `https://physoc.onlinelibrary.wiley.com/doi/abs/10.14814/phy2.13879`
11. Sobie, E.A.: Parameter sensitivity analysis in electrophysiological models using multivariable regression. Biophysical Journal **96**(4), 1264–1274 (2009). DOI 10.1016/j.bpj.2008.10.056
12. Srivastava, A., Valkov, L., Russell, C., Gutmann, M.U., Sutton, C.: Veegan: Reducing mode collapse in GANs using implicit variational learning. In: Advances in Neural Information Processing Systems, pp. 3308–3318 (2017)
13. Wolff Matthew R., McDonald Kerry S., Moss Richard L.: Rate of Tension Development in Cardiac Muscle Varies With Level of Activator Calcium. Circulation Research **76**(1), 154–160 (1995). DOI 10.1161/01.RES.76.1.154
